# Comprehensive Management of Maxillofacial Projectile Injuries at the First Operation; “Picking up the Pieces”

**DOI:** 10.5812/traumamon.9279

**Published:** 2013-01-15

**Authors:** Mohammad Hosein Kalantar Motamedi

**Affiliations:** 1 Department of Oral and Maxillofacial Surgery, Trauma Research Center, Baqiyatallah Medical Sciences University, Tehran, IR Iran

**Keywords:** Early Intervention (Education), Face, Skeleton, Firearms, Wounds and Injuries

The issue of when to treat maxillofacial firearm injuries with regard to timing (early or delayed), remains an issue of debate (1-4). Although not all maxillofacial projectile injuries can be comprehensively treated at onset, many indeed may (1-4). In over 50 cases of firearm injuries to the face at our center, more than 30 were treated via early comprehensive repair and simultaneous open reduction for maxillofacial fracture fixation. These patients underwent primary debridement and arch bar placement followed by open reduction of fractures (with or without osteosynthesis) followed by primary wound closure.

**Figure 1:**
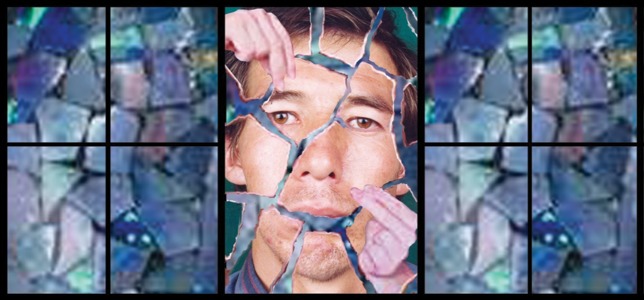
“Picking up the Pieces” and Putting Together a Mosaic Puzzle; a Similitude to Maxillofacial Reconstruction

Primary comprehensive intervention was done when there was no gross infection, no bone comminution, no extensive soft tissue avulsion (preventing wound coverage), and when general health, concomitant injuries requiring more urgent attention, or those requiring major grafts did not preclude this. Primary management included extensive oral and extraoral irrigation (dilute hydrogen peroxide + povidone iodide), brushing, debridement of the facial wound, removal of floating fragments (teeth particles, debris, and shell fragments) excluding viable bone within the wound. Next, after access to the remaining bone stumps, we sought to find scattered bone segments; after that we put them back into place to restore bone continuity (especially in the mandible) similar to “Picking up the Pieces” and putting together a puzzle (Figure 1). This is often possible more often than not because the bone is usually shattered and scattered, rather than avulsed or pulverized (5).

Treatment in addition to arch bars, employed titanium miniplates, recon-plates or wire osteosynthesis when necessary. All wounds were then closed primarily (using local advancement flaps); all patients were placed on antibiotics (cephalosporin + aminoglycoside or ciprofloxacin). In the acutely-treated group, few patients had complications (such as scarring and wound discharge) (6, 7).

Early comprehensive intervention for projectile wounds to the face was effective in selected cases. Our main aim was to restore continuity of the mandible. The benefits include restoration of occlusion and continuity of the jaw, earlier fixation of luxated teeth, early return of function, prevention of segment displacement and tissue contracture, less scarring, and a decreased need for major bone graft reconstruction later on and shorter hospital stay. Those treated secondarily were only debrided and had arch bars placed until later; the drawbacks of delaying include: Definitive treatment of hard and soft tissue management being rendered in another subsequent operation, bone reduction more difficult because of scarring, displacement of remaining segments (i.e. mandibular ramus), longer hospital stay and greater patient anxiety (8, 9). Additionally, if scattered bone pieces are not searched for in the wound and not reduced, a defect remains and those displaced bone fragments round-off; some may be resorbed. 

In terms of infection or other major complications e.g. chronic osteomyelitis, no significant differences were noted between early or delayed management. Thus, today many cases of projectile injuries to the face may be treated definitively and acutely with procedures designed to repair both bone and soft tissue injuries simultaneously aiming to restore bony continuity, esthetics and function using the tissues at hand (especially in the mandible). Because then, many patients with residual defects can be treated more easily as out-patients. Early treatment is also advocated because the course of healing is not disrupted with another subsequent operation (in the same wound) and because it may also decrease anxiety and stress associated with injured patients with disfigured faces and nonfunctional jaws anticipating definitive treatment (9).
